# 580. Changes in Serotype Distribution of Invasive Pneumococcal Infections in South Korea Before and After the COVID-19 Outbreak

**DOI:** 10.1093/ofid/ofad500.649

**Published:** 2023-11-27

**Authors:** Da Yun Kang, Ye Eun Kim, Ki Wook Yun, Eun Hwa Choi

**Affiliations:** Department of Pediatrics, Seoul National University Children’s Hospital, Seoul, South Korea, seoul, Seoul-t'ukpyolsi, Republic of Korea; Seoul National University Children's Hospital, Seoul, Seoul-t'ukpyolsi, Republic of Korea; Seoul National University Children's Hospital, Seoul, Seoul-t'ukpyolsi, Republic of Korea; Seoul National University Children's Hospital, Seoul, Seoul-t'ukpyolsi, Republic of Korea

## Abstract

**Background:**

The introduction of extended-valency pneumococcal conjugate vaccine (PCV) since 2014 has changed the serotype distribution in South Korea. The COVID-19 pandemic in 2020 has changed the epidemiology of several infectious diseases significantly, involving the invasive pneumococcal disease (IPD).

**Methods:**

This study obtained sterile body fluid samples of IPD in patients under 19 years old from January 2014 to April 2023 in South Korea, involved in multihospital-based surveillance system. Serotype distribution and multilocus sequence typing were performed for each sample. Vaccine serotypes (VTs) and nonvaccine serotypes (NVTs) were classified. The characteristics of IPD were compared between the pre-COVID-19 period (January 2014 to December 2019) and post-COVID-19 period (January 2021 to April 2023).

**Results:**

A total of 230 isolates of *Streptococcus pneumoniae* from sterile body fluid samples were obtained. Among those, VTs accounted for 14.3% of cases (19A, 8.3%; 19F, 3.0%; and 6A, 1.3%), NVTs for 83.9% (10A, 23.0%; 15A, 9.1%; 15C, 7.8%; 15B, 6.1%; 23B, 6.1%), and four cases (1.7%) were nontypeable. Serotype 10A decreased to 14.6% in the post-COVID-19 period, which was the most common serotype in the pre-COVID-19 period (25.4%). Among the NVTs, isolates of serotype 6D, 7C, 11A, 12F, 13, 20, 22F, 33F, 37, and 38 were not found in the post-COVID-19 period, although they accounted for 18.5% in the pre-COVID-19 period. Meanwhile, serotypes 6C, 15B/C, and 23B increased from 0.6%, 12.7%, and 2.9% in the pre-COVID-19 period to 8.3%, 18.8%, and 16.7% in the post-COVID-19 period, respectively. All the serotype 6C isolates found in the post-COVID-19 period were sequence type 13556. 66.7% of serotype 15B/C and 87.5% of serotype 23B in the post-COVID-19 period showed clonal complex 166.

**Conclusion:**

The COVID-19 pandemic has resulted in a shift in the predominant serotypes of invasive pneumococcal infections, with an increase in serotypes 6C, 15B/C, and 23B, which seems to be related with a certain clonal expansion. Notably, the cross-protection conferred by the PCV against serotype 6A appears to be insufficient for serotype 6C.

**Disclosures:**

**All Authors**: No reported disclosures

